# Evaluation of waist-to-height ratio as a predictor of insulin resistance in non-diabetic obese individuals. A cross-sectional study

**DOI:** 10.1590/1516-3180.2016.0358280417

**Published:** 2017-11-06

**Authors:** Giovana Jamar, Flávio Rossi de Almeida, Antonio Gagliardi, Marianna Ribeiro Sobral, Chao Tsai Ping, Evandro Sperandio, Marcelo Romiti, Rodolfo Arantes, Victor Zuniga Dourado

**Affiliations:** I MSc. Doctoral Student, Department of Biosciences, Universidade Federal de São Paulo (Unifesp), Santos, São Paulo (SP), Brazil.; II PT, MSc. Doctoral Student, Department of Human Movement Sciences, Universidade Federal de São Paulo (Unifesp), Santos, São Paulo (SP), Brazil.; III MD, PhD. Researcher, Department of Cardiovascular Medicine, Angiocorpore Instituto de Medicina Cardiovascular, Santos, São Paulo (SP), Brazil.; IV BM. Master’s Student, Postgraduate Program on Food, Nutrition and Health, Federal University of São Paulo (Unifesp), Santos, São Paulo (SP), Brazil.; V Physiotherapist, Universidade Federal de São Paulo (Unifesp), Santos, São Paulo (SP), Brazil.; VI PT, PhD. Associate Professor, Laboratory of Epidemiology and Human Movement, Universidade Federal de São Paulo (Unifesp), Santos, São Paulo (SP), Brazil.; VII PT, PhD. Associate Professor, Department of Human Movement Sciences, and Managing Professor, Laboratory of Epidemiology and Human Movement, Universidade Federal de São Paulo (Unifesp), Santos, São Paulo (SP), Brazil.

**Keywords:** Anthropometry, Obesity, Diabetes mellitus

## Abstract

**BACKGROUND:**

Insulin resistance (IR) and progressive pancreatic β-cell dysfunction have been identified as the two fundamental features in the pathogenesis of obesity and non-insulin-dependent diabetes mellitus. We aimed to investigate correlations between anthropometric indices of obesity and IR in non-diabetic obese individuals, and the cutoff value from receiver operating characteristic (ROC) curve analysis.

**DESIGN AND SETTING:**

Cross-sectional study conducted in a private clinic.

**METHODS:**

We included obese individuals (body mass index, BMI ≥ 30 kg/m^2^) with no diabetes mellitus (fasting glucose levels ≤ 126 mg/dl). The participants were evaluated for the presence of cardiovascular risk factors and through anthropometric measurements and biochemical tests. Furthermore, IR was assessed indirectly using the homeostatic model assessment (HOMA)-IR and HOMA-β indexes. The area under the curve (AUC) of the variables was compared. The sensitivity, specificity and cutoff of each variable for diagnosing IR were calculated.

**RESULTS:**

The most promising anthropometric parameters for indicating IR in non-diabetic obese individuals were waist-to-height ratio (WHtR), waist circumference (WC) and BMI. WHtR proved to be an independent predictor of IR, with risk increased by 0.53% in HOMA-IR, 5.3% in HOMA-β and 1.14% in insulin. For HOMA-IR, WHtR had the highest AUC value (0.98), followed by WC (0.93) and BMI (0.81). For HOMA-β, WHtR also had the highest AUC value (0.83), followed by WC (0.75) and BMI (0.73). The optimal WHtR cutoff was 0.65 for HOMA-IR and 0.67 for HOMA-β.

**CONCLUSION:**

Among anthropometric obesity indicators, WHtR was most closely associated with occurrences of IR and predicted the onset of diabetes in obese individuals.

## INTRODUCTION

Insulin resistance (IR) is considered to be one of the main risk factors for cardiovascular disease (CVD). It is associated with several metabolic abnormalities such as impaired glucose tolerance, non-insulin-dependent diabetes mellitus (NIDDM), hypertension and dyslipidemia.^1^^,^^2^ Maintenance of normal blood glucose comes mainly from the ability of β-pancreatic cells to secrete insulin and the sensitivity of the target tissues to respond to normal levels of insulin in the bloodstream.^3^

The homeostasis model assessment (HOMA) is a widely validated clinical and epidemiological tool for estimating IR and β-cell function. It is derived from a mathematical assessment of the balance between hepatic glucose output and insulin secretion from fasting levels of glucose and insulin.^4^ HOMA-IR and HOMA-β have been adopted as an alternative to the gold standard method, i.e. the hyperinsulinemic-euglycemic clamp technique. Although use of HOMA indices requires an invasive access,^5^ it is inexpensive and easy to apply.^6^

One aspect of research on obesity that is currently attracting attention is the distribution of fat in the body. Diabetes, atherosclerosis and sudden cardiac death occur quite frequently among obese people, but when obesity is centralized in the abdominal region, the negative repercussions (both metabolic and cardiovascular) are more significant.^7^ Several studies have evaluated the correlation between IR and anthropometric indices of obesity such as body mass index (BMI), waist circumference (WC), neck circumference (NC) and hip circumference (HP). They have demonstrated that the distribution of visceral fat causes significant damage to the insulin-signaling pathway due to secretion of adipokines, e.g. C-reactive protein (CRP),^2^^,^^8^^,^^9^ thus leading to increased cardiometabolic risk.^10^ Therefore, obesity is the most prominent predictor of IR and diabetes.^11^

Anthropometry is considered to be a non-invasive tool for early diagnosis of the onset of NIDDM. In addition, it provides an alternative evaluation of IR at lower cost that is accessible for application in epidemiological studies and primary care within health services.^8^ However, there is no consensus regarding which anthropometric measurement is most indicative of IR in non-diabetic obese subjects, or regarding the cutoff values.

## OBJECTIVE

We aimed to investigate the correlations between anthropometric indices of obesity and IR in non-diabetic obese individuals, and to identify the best cutoff values of these indices for predicting IR, through using receiver operating characteristic (ROC) curve analyses.

## METHODS

### Participants

This study used a cross-sectional design. The participants were selected as a convenience sample of consecutive patients admitted between 2013 and 2015, when they presented the following inclusion criteria: BMI ≥ 30 kg/m^2^ and no diabetes mellitus (DM) (reported or fasting blood glucose ≤ 126 mg/dl).^12^^,^^13^ We enrolled 136 obese individuals, comprising 72 men and 64 women, at the Obesity Clinic of the Angiocorpore Institute of Cardiovascular Medicine, located in the city of Santos, São Paulo, Brazil. They had been referred for the examinations because of a variety of medical indications. This study formed part of a larger study assessing the determinants of exercise intolerance among obese individuals. All the participants agreed to participate, and none of them presented abnormalities during the examinations that would exclude them.

The Ethics Committee for Research on Human Beings of the Federal University of São Paulo (Universidade Federal de São Paulo, UNIFESP) approved this study under the number 1.079.239. Furthermore, an informed consent statement was signed by all of these volunteers.

### Anthropometric obesity indices

Body weight and height were measured by using a weighing scale with stadiometer that measured to precisions of the nearest 0.1 kg and 1 cm (Toledo, São Paulo, Brazil). The individuals were weighed without shoes. The neck (NC), waist (WC) and hip (HC) circumferences were measured in cm using an inelastic tape (Sanny) with precision of 1 mm. We measured NC at the midpoint of the neck; WC at the midpoint between the last rib and the iliac crest; and HC at the point of greatest gluteal protuberance.^14^^,^^15^ From these anthropometric measurements, we obtained indices relating to cardiometabolic health: waist-to-hip ratio (WHR), waist/height ratio (WHtR), body mass index (BMI = weight_kg_/height_m²_) and body shape index (BSI = WC/BMI^2/3^ x height^½^).^16^

### Blood test

Blood samples were collected for laboratory-based biochemical measurements after the participants had fasted for 12 hours. We quantified C-reactive protein (CRP, ng/ml), total cholesterol (mg/dl), HDL cholesterol (mg/dl), LDL cholesterol (mg/dl), insulin (IU/dl) and glucose (mg/dl). Glucose values were converted from mg/dl to mmol/l using the conversion factor 0.555.^13^

### IR assessment

We used the homeostasis model assessments HOMA-IR and HOMA-β to indirectly determine IR, based on glucose and insulin values proposed by Matthews et al.^3^ IR was defined as situations with HOMA-IR ≥ 2.7,^17^^,^^18^^,^^19^^,^^20^^,^^21^ and dysfunction of β-cells as situations with HOMA-β > 175.^4^^,^^22^

### Cardiovascular risk assessment

We assessed self-reported cardiovascular risk factors in accordance with the recommendations of the American College of Sports Medicine (ACSM). The participants were asked to report any previous diagnosis of the main cardiovascular risk factors such as arterial hypertension, dyslipidemia and diabetes, along with their age, situation of physical inactivity and smoking status. We considered that the participants were physically inactive if they reported doing less than 150 minutes per week of moderate-to-vigorous physical activity.^23^

### Statistical analysis

We assessed correlations between anthropometric indices and HOMA-IR values, HOMA-β values and insulin concentration using Pearson correlation coefficients. Three models of stepwise multiple linear regressions were then fitted, with HOMA-IR, HOMA-β and insulin as the main outcomes. The main predictors that we chose were the anthropometric indices that significantly correlated with outcomes after univariate analysis. We checked for multicollinearity in the models by means of variation inflation factor (VIF) values < 4. The models were also adjusted for age, sex and cardiovascular risk factors.

We fitted ROC curves to assess the best cutoff points for anthropometric measurements for predicting clinically high values of HOMA-IR and HOMA-β as surrogate measurements for IR. The areas under the ROC curves (AUC) and the 95% confidence intervals (95% CI) were used to compare the diagnostic value of various obesity indices. We considered that values above 0.80 were excellent. The main anthropometric indices selected after multiple linear regression were used to obtain the optimal cutoff point for diagnosing IR. We calculated the sensitivity, specificity, positive and negative likelihood ratios and Youden index in relation to these values.

All tests were evaluated at a two-tailed alpha level of 0.05. All statistical analyses were performed using the Statistical Package for the Social Sciences (SPSS), version 23 (SPSS Inc., Chicago, USA), and the MedCalc package, version 17 (MedCalc Software bvba, Belgium).

## RESULTS

### Baseline characteristics of the participants

The men and women involved in the present study were on average middle-aged. We found significantly higher values for weight, height, WC, WHR, NC and BSI among the men, while HC and BMI were significantly higher among the women. The participants were mostly physically inactive. We observed a greater proportion of dyslipidemia among the men and higher fasting glucose among the women (Table 1).

**Table 1.**
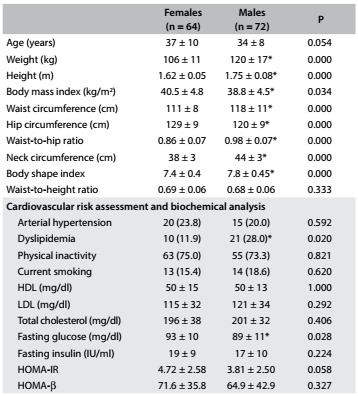
General characteristics of the study sample according to sex. Mean ± standard deviation (SD).

HDL = high-density lipoprotein; LDL = low-density lipoprotein; HOMA-IR = homeostasis model assessment - insulin resistance; HOMA-β = homeostasis model assessment - beta-cell function. *P < 0.05 = females versus males.

When stratified according to nutritional status, we found progressively impaired values for fasting insulin, HOMA-IR and HOMA-β with increasing severity of obesity, while CRP presented a significant difference only at obesity level I and total cholesterol at obesity level III (Table 2).

**Table 2.**
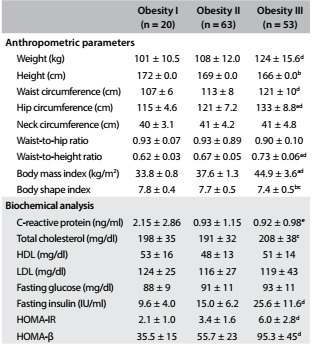
Description of anthropometric measurements and biochemical analysis between obesity levels. Mean ± standard deviation (SD).

HDL = high-density lipoprotein; LDL = low-density lipoprotein; HOMA-IR = homeostasis model assessment - insulin resistance; HOMA- β = homeostasis model assessment - beta-cell function. a = obesity I versus obesity II, P < 0.05. b: obesity I versus obesity III, P < 0.05. c = obesity II versus obesity III, P < 0.05. d = obesity I and obesity II versus obesity III, P < 0.05. e: obesity II and obesity III versus obesity I, P < 0.05.

### Correlation and multiple regression analysis

We found strong correlations of WHtR, WC and BMI with HOMA-IR, HOMA-β and fasting insulin. On the other hand, WHR, NC and BSI showed weak correlations (Table 3). A stepwise multiple linear regression analysis was performed with HOMA-IR, HOMA-β and insulin as dependent variables. After adjustment for age, sex and obesity indices, WHtR proved to be an independent predictor of IR in this study (Table 4).

**Table 3.**
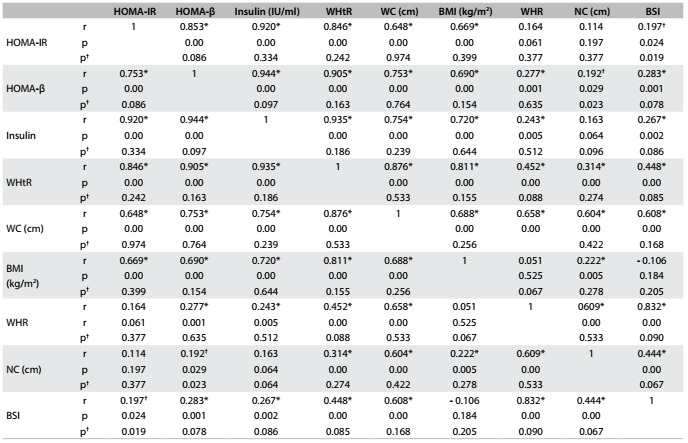
Matrix of correlations between obesity indices and values for homeostasis model assessment - insulin resistance (HOMA-IR) and for HOMA - beta-cell function (HOMA-β) in the study sample

r = Pearson coefficient; WHtR = waist-to-height ratio; WC = waist circumference; BMI = body mass index; WHR = waist-to-hip ratio; NC = neck circumference; BSI = body shape index. *Significant correlations for all subjects; ^†^Significant correlations for females versus males.

**Table 4.**
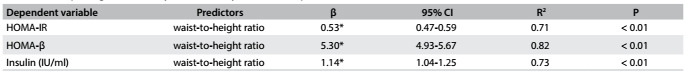
Multiple regression analysis on obesity indices that predict insulin resistance

WHtR = waist-to-height ratio; HOMA-IR = homeostasis model assessment - insulin resistance; HOMA-β = homeostasis model assessment - beta-cell function. *WHtR values are expressed in percentage. Models adjusted for age, sex, weight, waist circumference, neck circumference, body mass index, waist-hip ratio, waist-to-height ratio and body shape index.

### ROC curves

**Figure 1.**
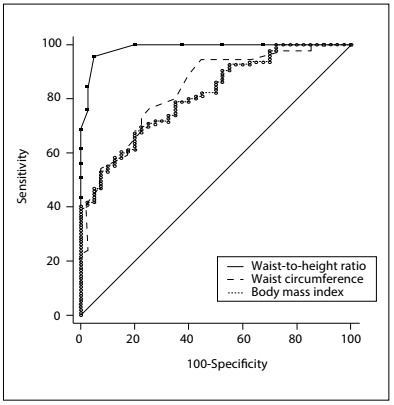
Receiver operating characteristic (ROC) curve for anthropometric parameters that predict insulin resistance according to the homeostatic model assessment-insulin resistance (HOMA-IR). The areas under the ROC curves and the 95% confidence intervals (95% CI) were 0.98 (0.95-0.99) for waist-to-height ratio (WHtR); 0.93 (0.76-0.89) for waist circumference (WC); and 0.81 (0.74-0.87) for body mass index (BMI).

**Figure 2.**
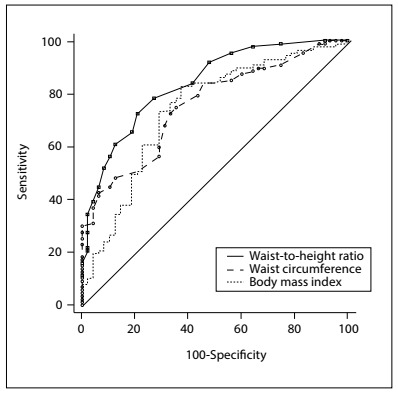
Receiver operating characteristic (ROC) curve for anthropometric parameters that predict insulin resistance according to the homeostatic model assessment (HOMA)-β. The areas under the ROC curves and the 95% confidence intervals (95% CI) were 0.83 (0.76-0.89) for waist-to-height ratio (WHtR); 0.75 (0.67-0.82) for waist circumference (WC); and 0.73 (0.65-0.81) for body mass index (BMI).

The abilities of WHtR, WC and BMI to detect IR were compared using ROC curves. For HOMA-IR, we found an AUC of 0.98 for WHtR, 0.83 for WC and 0.81 for BMI, such that the AUC was significantly greater for WHtR than for WC (difference between areas = 0.150; P < 0.001) and BMI (difference between areas = 0.171; P < 0.001). We found that there was no significant difference in AUC between WC and BMI (difference between areas = 0.021; P = 0.629) (Figure 1). Regarding HOMA-β, the AUC of 0.83 for WHtR was significantly greater than the AUC for WC (0.75, difference between areas = 0.082; P = 0.013) and BMI (0.73, difference between areas = 0.099; P = 0.009), with no significant difference between WC and BMI (difference between areas = 0.017; P = 0.727) (Figure 2). The best cutoff points for HOMA-IR were 0.65, 113 cm and 38.76 kg/m^2^ and for HOMA-β were 0.67, 112 cm and 37.61 kg/m^2^, respectively for WHtR, WC and BMI (Table 5).

**Table 5.**
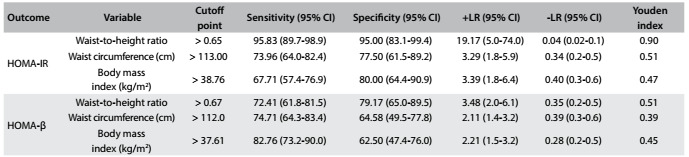
Optimal cutoff point values and their related sensitivity, specificity, positive and negative likelihood ratios and Youden index for obesity indices, regarding discrimination of insulin resistance

HOMA-IR = homeostasis model assessment - insulin resistance; HOMA-β = homeostasis model assessment - beta-cell function; +LR = positive likelihood ratio; -LR = negative likelihood ratio.

## DISCUSSION

In the present study, we observed that not all the anthropometric parameters studied were significantly associated with HOMA-IR and HOMA-β. The most promising anthropometric parameters for indicating IR in non-diabetic obese adults were WHtR, WC and BMI. Our results suggest that there are advantages to using WHtR. In our analysis, we observed that the risk of IR was raised by 0.53% in HOMA-IR, 5.3% in HOMA-β and 1.14% in insulin for each additional 1% increase in WHtR (= 0.01). Thus, WHtR was a predictor for the degree of IR and predisposition towards diabetes in our sample of obese individuals. Recently, Vikam et al.^10^ observed increased odds ratios for hyperinsulinemia and metabolic syndrome among individuals with WHtR > 0.5.

Use of WHtR for detecting abdominal obesity and its associated risks to health was first proposed in the 1990s.^24^ The growing body of literature showed that this abdominal obesity indicator could predict the cardiometabolic risk even better than BMI and WC.^25^ A recent meta-analysis on studies evaluating different indices of adiposity showed that WHtR was a better predictor for hyperinsulinemia, diabetes, arterial hypertension, dyslipidemia, metabolic syndrome and other cardiovascular health problems than were BMI or WC, in both men and women.^26^ In addition, our AUC values for this anthropometric obesity indicator were higher than in previous prediction studies with WHtR,^11^^,^^27^^,^^28^ thus emphasizing the accuracy of AUC measurements for identifying IR in obese populations. According to Behboudi-Gandevani et al.,^11^ WHtR may be proposed as a sensitive, inexpensive, noninvasive, simple-to-assess and easy-to-calculate tool for screening for IR.

Taking into account that ethnicity and gender may influence body composition, studies on Brazilian and Indian overweight women also showed that the WHtR was the most important predictive measurement for IR and diabetes.^27^^,^^29^ However, studies on men of different ethnicity indicated that BMI was the best predictor for IR.^28^^,^^30^^,^^31^ It should be noted that BMI is characterized as an indicator of general adiposity because of its inability to assess the distribution of body fat, thus presenting a weaker relationship with visceral fat.^27^ In a recent meta-analysis, Savva et al.^32^ compared the association of BMI and WHtR with the cardiometabolic risk factor of diabetes in Asian and non-Asian populations. The data from cross-sectional studies indicated that WHtR is superior to BMI for detecting diabetes in both Asian and non-Asian populations. There are still few studies of this design on Brazilian populations, especially in relation to obese individuals.^32^

The risk of developing obesity-related comorbidities is proportional to the degree of obesity and, more specifically, the accumulation of visceral fat.^33^ However, the presence of metabolic disorders varies considerably among obese individuals,^34^ since it is known that there is one subgroup of obese individuals that seems to be protected against or is more resistant to developing cardiometabolic complications.^35^ Nevertheless, regarding phenotypes for metabolic status and diabetes, healthy obese and metabolically unhealthy normal-weight individuals appear to have an equivalent risk.^36^

In the general population, a WHtR cutoff < 0.5 is recommended, which can be presented as a simple public health message that individuals should seek to maintain their WC as less than half of their height. We showed that the higher this ratio is, the higher the risk of indirect IR is, and we proposed a cutoff > 0.65 to identify IR in non-diabetic obese individuals. This indicates that there is a need for a specific evaluation on this population, for early detection of IR that could ultimately reduce the incidence or severity of diabetes and cardiovascular diseases.

In summary, we found that WHtR may be useful in clinical practice due to its advantageous simplicity. Also, it is easy to calculate, does not require any special equipment other than an inelastic tape, and only requires some rater training.

The present study has limitations that should be considered. Our sample was not enough to extract the cutoff points according to sex. Since not all obese individuals have metabolic alterations, our strategy was to ascertain which anthropometric measurements were better correlated with IR, and whether non-diabetic obese individuals would present a cutoff point different from general population for predicting the onset of diabetes, thereby suggesting different reference values for a more accurate assessment in this specific group. Perhaps inclusion of a eutrophic group would have contributed towards reinforcing our important findings. Future research should aim to screen Brazilian obese populations, in order to provide support for our remarks.

## CONCLUSION

We can conclude that the WHtR is a strong predictor of IR, as assessed using HOMA, among non-diabetic obese adults. Our results suggest that WHtR can form a simple and powerful tool for screening for IR among these patients, since we found convincing AUC and sensitivity and specificity values for this index in detecting clinically high values of HOMA-IR and HOMA-β.
